# Pulsed Photothermal Radiometric Depth Profiling of Bruises by 532 nm and 1064 nm Lasers

**DOI:** 10.3390/s23042196

**Published:** 2023-02-15

**Authors:** Ana Marin, Rok Hren, Matija Milanič

**Affiliations:** 1 Faculty of Mathematics and Physics, University of Ljubljana, 1000 Ljubljana, Slovenia; 2 Institute of Mathematics, Physics, and Mechanics, 1000 Ljubljana, Slovenia; 3 Jozef Stefan Institute, 1000 Ljubljana, Slovenia

**Keywords:** pulsed photothermal radiometry, depth profiling, bruise, KTP, Nd:YAG

## Abstract

Optical techniques are often inadequate in estimating bruise age since they are not sensitive to the depth of chromophores at the location of the bruise. To address this shortcoming, we used pulsed photothermal radiometry (PPTR) for depth profiling of bruises with two wavelengths, 532 nm (KTP laser) and 1064 nm (Nd:YAG laser). Six volunteers with eight bruises of exactly known and documented times of injury were enrolled in the study. A homogeneous part of the bruise was irradiated first with a 5 ms pulse at 532 nm and then with a 5 ms pulse at 1064 nm. The resulting transient surface temperature change was collected with a fast IR camera. The initial temperature–depth profiles were reconstructed by solving the ill-posed inverse problem using a custom reconstruction algorithm. The PPTR signals and reconstructed initial temperature profiles showed that the 532 nm wavelength probed the shallow skin layers revealing moderate changes during bruise development, while the 1064 nm wavelength provided additional information for severe bruises, in which swelling was present. Our two-wavelength approach has the potential for an improved estimation of the bruise age, especially if combined with modeling of bruise dynamics.

## 1. Introduction

Bruises are skin lesions most commonly caused by blunt force trauma when small vessels burst and a blood pool forms between the dermal and subcutaneous layer. To detect and hopefully prevent physical abuse, it is of utmost importance to monitor the development of bruises, especially in children, women, and older adults [1,2]. Nowadays, the age of bruises is still mostly determined by the clinician’s visual inspection based on the color of the bruise, which is often quite subjective, inaccurate, and unreliable [3,4] due to factors such as ambient lighting, skin thickness, and depth of the bruise.

The search for a more objective technique led to the use of bilirubinometry, colorimetry, and L*a*b* color space [5,6,7,8,9]; however, the information obtained from only a few color channels is severely limited. By measuring the spectrum, diffuse reflectance spectroscopy (DRS) [10,11] provides additional information about specific chromophores and their concentrations in the skin. An obvious expansion of reflectance spectroscopy is multispectral or hyperspectral imaging (HSI), which furnishes additional spatial information. In HSI modality, an image is generated in which each pixel contains spectral information of a broad spectrum of wavelengths. HSI has become a promising technique in evaluating bruises since it considers the bruise shape and the spatial distribution of chromophores [12,13,14,15,16]. 

However, these techniques are not sensitive to the chromophore depth distribution, as scattered light from many tissue layers is collected and analyzed indiscriminately. An attractive alternative is pulsed photothermal radiometry (PPTR), which has sufficient penetration depth and the ability to determine the depth distribution of chromophores [17,18,19,20,21]. In PPTR, we specifically measure transient changes in mid-infrared emissions from the sample surface after exposure to a short-light pulse; these signals are then used to noninvasively assess irradiation-induced temperature–depth profiles to reveal the depths of absorbing structures [22,23]. This technique enables the localization of selected chromophores within biological tissue and has been successfully applied in tissue phantoms [24,25], healthy skin [26], and port wine stains [27,28]. KTP lasers have been used [29,30,31] to determine blood spillage in bruises, while the near-infrared (NIR) wavelengths have been applied to investigate energy deposition profiles in healthy skin [32]. Characteristics of colorimetry, DRS, HSI, and PPTR are comprehensively compared in Table 1.

In this article, we introduce PPTR profiling of bruises that combined two wavelengths, 532 nm from the KTP laser and 1064 nm from the Nd:YAG laser. A dual-wavelength PPTR method was first reported by Majaron et al. [18], who were able to separate the superficial vascular and epidermal components of the PPTR signal using 585 nm and 600 nm lasers. However, the reported wavelengths have small penetration depths in human skin. To probe deeper skin regions where a blood pool in bruises typically resides, a different combination of wavelengths must be employed. We selected the 1064 nm light which penetrates several mm into the tissue, and the 532 nm light, which penetrates only several hundred μm [35] but is selectively absorbed in superficial skin blood and melanin. Since the tissue optical properties at the two utilized laser wavelengths differed substantially, we obtained additional information about the chronological evolution of chromophore distributions in bruised skin. Specifically, we systematically present chronological changes of eight accidental traumatic bruises that occurred at different sites on the body. The 532 nm/1064 nm approach thus provides novel information about bruise dynamics obtained in vivo, which cannot be obtained by other modalities.

## 2. Materials and Methods

As already mentioned, bruises form due to small vessels bursting and a blood pool forming between the dermal and subcutaneous layer. After the pool is formed, visual changes can be observed as the hemoglobin contained in the blood diffuses toward the epidermis. At the same time, hemoglobin is broken down by heme-oxygenase and transformed first into biliverdin and next rapidly into bilirubin, the main chromophore responsible for the yellowish color of old bruises. These waste products are then gradually removed through the lymphatic drain [36] until the bruise has subsided, which usually occurs two weeks after the injury.

The PPTR technique and its theoretical background have been described in detail elsewhere [17]. It is based on utilizing temporal heat diffusion information, where heat waves originating from heated parts of a sample diffuse toward the sample surface. Briefly, PPTR measures the transient change in infrared (IR) tissue emission after laser irradiation Δ*S*(*t*):

(1) $$\Delta S(t)=\int_{0}^{\infty }K(z,t)\Delta T(z,0)dz,$$

where kernel function *K*(*z*,*t*) accounts for skin thermal properties and an effective IR absorption coefficient. Specifically, *K*(*z*,*t*) depends on the skin thermal diffusion constant, which was 0.11 mm^2^/s, the reduced heat transfer coefficient being 0.02 mm^−1^, and the effective IR absorption coefficient value for the 3.0–5.6 µm detection spectral band used in the study. The effective IR absorption coefficient of 22.3 mm^−1^ corresponding to the large spectral variation of the skin attenuation coefficient μ(λ) in the mid-IR was calculated as described in [23].

Δ*T*(*z*,0) represents the initial laser-induced temperature profile. By representing ΔS and Δ*T* as vectors ***S*** and ***T***, and ***K*** as a matrix, Equation (1) can be formulated as a set of linear equations:

(2) $$S=\mathrm{KT};K_{i,j}=K(z_{j},t_{i})\Delta z.$$

However, due to the defect rank of ***K*** and experimental noise, reconstruction of ***T*** from ***S*** presents a severely ill-posed problem [17]. In general, the temperature profile can be reconstructed by iterative minimization of the squared residual norm ||***S*** − ***KT***||^2^ and applying a non-negativity constraint. In this study, a custom reconstruction algorithm based on the projected ν-method [23] was used. This theory was valid for sufficiently wide irradiation so that laterally homogenous heat distribution could be assumed (1D approximation). The axial resolution of the reconstructed profile was nonlinear and decreased with depth [23].

Six volunteers (five females and one male) were enrolled in the study, four with one bruised site and two with two bruised sites (eight bruises in total). The volunteers were between 22 and 29 years old, had fair skin (Fitzpatrick scale type I–II), and no history of blood clotting problems; none were smokers or vegetarians. The bruising was incidental caused by falls, bumps, and sport accidents, with the exact time of the injury duly reported. Only sites with an intact skin surface and a homogeneous part of the bruise with an area of at least 1 cm^2^ were considered for this study. Informed consent was obtained from each of the volunteers, and the procedure was performed in accordance with the Declaration of Helsinki. The study protocol was approved by the Medical Ethics Committee of the Republic of Slovenia (111/02/12).

The measurements were performed four to seven times on the bruised site and on a nearby uninjured location with a presumably similar skin composition, which served as a reference. At the beginning, the measurements were performed daily or every other day, while the interval between measurements increased as the bruise healed (up to an additional five days) since the bruise was changing more slowly later in the healing process.

Prior to the measurement, the skin at the test site was shaved, while the superficial layer of dehydrated skin cells from the stratum corneum was removed by tape stripping to ensure unobstructed heat diffusion. The site was then cleaned with medical ethanol to remove any glue residue, rehydrated with a physiological solution, and left to dry.

The test sites were first irradiated with 5 ms pulses at 532 nm, followed by 5 ms pulses at 1064 nm, with both wavelengths emitted from a medical-grade laser (DualisVP, Fotona, Slovenia). The effective spot size was approximately 9 mm, and the radiant exposures were 0.18–0.22 J/cm^2^ for 532 nm and 0.9–1.1 J/cm^2^ for 1064 nm laser. The angle of incidence for both lasers was 17°.

The mid-IR emission from the skin surface was recorded by an IR camera (SC7500, FLIR Systems Inc., Wilsonville, OR, USA, spectral range 3.5–5.1 μm) at a rate of 1000 frames per second. The radiometric signals Δ*S*(*t*) were determined by laterally averaging the recorded images over a sub-window corresponding to an area up to about 8 mm^2^ and subtracting the baseline temperature. The location of the sub-window was chosen to avoid hot spots—areas of increased heating due to hair follicles or other strong skin absorbers. The calibration system provided by the manufacturer (HypercalTM) was used for the nonlinear conversion of the radiometric signals to radiometric temperatures. From three to five measured radiometric temperature signals were used to obtain an average signal.

The initial temperature profiles ***T*** for both irradiation wavelengths were reconstructed from the average signals ***S*** using Equation (2) and the projected ν-method [23]. The Equation (2) parameters, including the effective IR attenuation coefficient, heat diffusion constant, and heat transfer coefficient, were the same for both wavelengths. The profiles consisted of 400 equidistant temperature values over a depth range of 1.5 mm or 2.0 mm for 532 nm pulses, with more severe bruises requiring shallower ranges (see bruise #3). The profiles for 1064 nm pulses were extended to 3.0 or 3.5 mm as a result of a higher penetration depth. 

To quantitatively compare the reconstructed temperature profiles, the integral under the curve (IUC) was calculated for each profile using the trapezoidal rule for integration. The IUC was a good estimate of how much of the laser energy was absorbed in the tissue during the laser pulse. Bruises were expected to have increased energy absorption compared with healthy skin since the blood pool provides additional absorbers arising from extravasated hemoglobin and water in the case of swelling. The ratio between the IUC of bruised and healthy skin, *ε*, was calculated as

(3) $$\varepsilon =\frac{{\mathrm{IUC}}_{\mathrm{bruise}}}{{\mathrm{IUC}}_{\mathrm{healthy}}}-1$$

Before applying the experimental protocol in the study, we considered the impact of the signal acquisition time of the deep-penetrating NIR laser on inversely reconstructed temperature profiles. If the signal acquisition time was too short, the information about deeper structures might not be recorded, and if it was too long, the signal might be affected by various artifacts, such as subject motion, temperature change due to ambient air, changes in blood flow, or autoregulation. To find the optimal signal acquisition time for the 1064 nm laser, a set of 10 s signals were collected on healthy and bruised skin. The reconstructed temperature profiles did not change considerably for times longer than 5 s. Therefore, the 5 s signal acquisition time was selected as the optimal signal length for the 1064 nm laser, while the 532 nm laser required a shorter signal acquisition time of 3 s.

## 3. Results

The Results section is organized as follows. For bruise #1, both the PPTR signals and the inversely computed initial temperature–depth profiles are presented, while for bruise #2 and bruise #3, only the initial profile reconstructions are shown and discussed; no initial temperature profile reconstructions are shown for bruises #4 through #8. The Results section is concluded by considering the IUC for bruises #1 through #8.

### 3.1. Bruise #1—PPTR Signals

**Figure 1:**
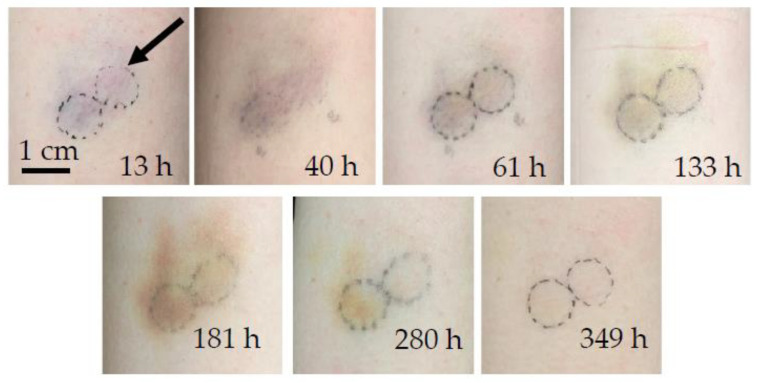
Chronological evolution of bruise #1. The arrow indicates the circular area of measurement. The typical bruise color changes described in [37] can be ascertained, from the bluish fresh bruise, with more pronounced redness a day later, to a brown coloration as bilirubin became prominent after the fourth measurement at 133 h and persisted as a yellow hue until the eventual disappearance of the bruise after 349 h (two weeks).

**Figure 2:**
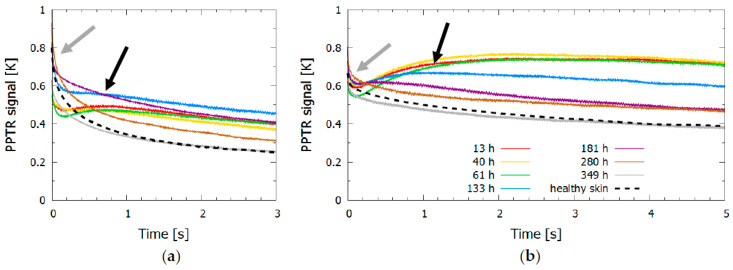
Time evolution of (**a**) KTP (532 nm) and (**b**) Nd:YAG (1064 nm) signals. The control signal for healthy skin is shown with a dashed line, and the signals for bruised skin at different times after the injury are shown in different colors; the gray arrow indicates the initial temperature increase due to shallow absorbers, and the black arrow indicates the temperature increase due to the blood pool forming deeper in the dermis.

The chronological evolution of bruise #1 is shown in Figure 1, and the corresponding PPTR signals are shown in Figure 2. The bruise occurred on the exterior of the left upper arm of a 29-year-old female during roller derby practice. Its evolution was monitored from 13 h (0.5 days) to 349 h (14.5 days) after the occurrence of an injury.

The dynamics of surface temperature change revealed for both lasers (Figure 2) an initial temperature increase due to shallow absorbers, mainly melanin in the epidermis (indicated by a gray arrow). The second temperature peak or slow thermal relaxation indicated heat accumulation from a blood pool forming at the time of the injury deeper in the skin, at the border between the dermis and subcutis; this second peak was progressively moving toward the surface with time after the injury due to hemoglobin molecules gradually diffusing through the dermal layers and eventually stopping at the epidermal-dermal junction.

For the 532 nm laser pulse, the first four measurements (up to 133 h after the injury) in Figure 2a showed heat accumulation deeper in the dermis as evident by a second increase in the signal temperature after 0.5 s (marked by a black arrow). After the fifth measurement (at 181 h) the surface temperature began to decrease more rapidly and approached the signal of healthy skin. The changes in the signals were indicative of the changes seen in Figure 1, where the bruise had almost disappeared at 280 h and 349 h after the injury. The signal from the healthy skin and the signal from the bruise obtained after the bruise had healed still differed slightly in shape and amplitude, as shown in Figure 2 (gray and dashed black line); however, the observed difference did not exceed 10% of the signal. The possible reasons for this difference included changes in skin constitution (e.g., changes in melanin concentration due to sun exposure, daily changes in skin hydration, or different scattering properties), which could slightly alter the shape of the signal, or variations in the laser fluence (up to 10%).

The signals of the 1064 nm laser pulse (Figure 2b) showed similar trends to those observed when using the 532 nm laser. However, the skin temperature after the injury decreased at a much slower rate, which was due to the heat accumulated deeper in the dermis; for example, the signal from the healthy skin decreased by 70% after 3 s for a 532 nm light pulse, whereas it decreased by only 35% for a 1064 nm light pulse. 

### 3.2. Bruise #1—Inversely Reconstructed Initial Temperature–Depth Profiles

**Figure 3:**
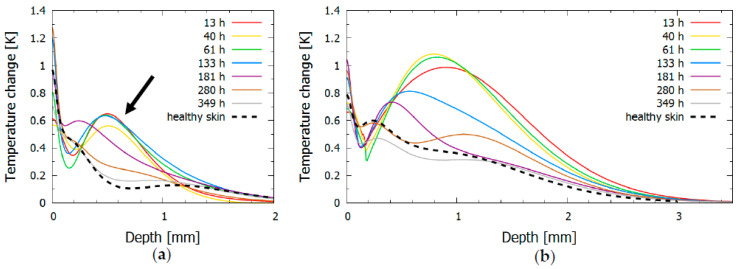
Inversely reconstructed initial temperature–depth profiles of bruise #1 for laser wavelengths (**a**) 532 nm and (**b**) 1064 nm. The profile for healthy skin is shown with a dashed line, and profiles for bruised skin at different times after the injury are shown in different colors; the black arrow indicates a prominent temperature peak, which was not present in the healthy skin.

The PPTR signals (Figure 2) were used to inversely reconstruct the initial temperature profiles in the bruised and healthy skin (Figure 3). The profiles induced by the 532 nm laser (Figure 3a) showed two distinct temperature peaks in the bruised skin and three peaks in the healthy skin. The narrow superficial peak in the outermost 100 μm of the skin was due to melanin absorption in the epidermis, while the broader deeper peaks resulted from either absorption in the vessels (papillary or upper blood net dermis) or hemoglobin diffusing from the blood pool.

In the bruised skin, a prominent peak at approximately 0.5 mm (black arrow in Figure 3a), which was not present in the healthy skin, indicated an accumulated traumatic blood pool, as hemoglobin was the most prominent skin absorber at 532 nm. With time passed after the injury, the peak slowly moved toward the surface and began to decrease after one week, eventually disappearing as the extravasated hemoglobin and its metabolic products were removed from the skin. However, this peak did not represent the entire blood pool as the 532 nm light could only probe the most superficial dermal layers before being completely absorbed in the blood.

The amount of heat that accumulated in the epidermal peak also changed through time, being less pronounced in the first few days after the injury and more prominent after about a week. It was highly unlikely that this trend was due to changes in the melanin concentration, since the measurement was taken on a covered body part. One possible explanation was that the absorption of the blood pool in the first days reduced the amount of backscattered light. However, an increase in the papillary dermis blood volume or edema—due to the increased refractive index mismatch and hydration [38]—would have increased the scattering, resulting in more backscattered photons. The observed behavior was thus a combination of two opposing effects, with each dominating at a different time in the bruise development [22].

The 1064 nm light penetrated much deeper into the skin, as shown by the inversely reconstructed initial temperature–depth profiles in Figure 3b. The second peak was very pronounced and extended to depths greater than 2 mm. Its gradual move to shallower depths (a probable result of the bruise diffusing toward the surface) was more evident than with 532 nm light, as was the decrease in peak amplitude after 5 days. The shallow peak was also present in these profiles, indicating light absorption in epidermal melanin. At later times after injury (at 280 and 349 h), two peaks were present, which approached the skin profile of healthy skin. A peak at 0.25 mm most likely corresponded to papillary dermis and a peak deeper than 1 mm possibly corresponded to either a deep dermal blood net, a deep lying vein, or heated subcutaneous tissue. 

Both lasers provided information about bruise evolution. However, because the 1064 nm laser penetrated deeper into the tissue, it offered additional insight into the changes resulting from the bruise healing, which was not present in the 532 nm profiles. For example, the 1064 nm profile at 133 h showed a considerable change compared with the profile at 61 h, while the change in the 532 nm profile was not yet visible.

### 3.3. Bruise #2—Inversely Reconstructed Initial Temperature–Depth Profiles

**Figure 4:**
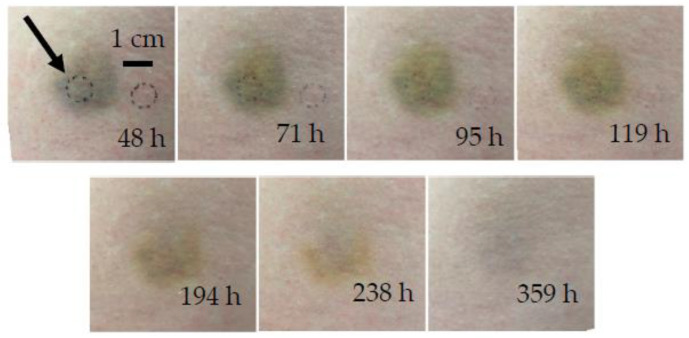
Chronological evolution of bruise #2. The arrow indicates the circular area of measurement.

Bruise #2 occurred under the left hip of a 29-year-old female due to colliding with a doorknob (Figure 4). Bruise #2 was measured seven times between 48 and 359 h (2 to 15 days) after injury. Photographs taken at 71 h, 95 h, and 119 h showed minimal differences in the color and shape of the bruise; from 194 h onward, the bruise degradation products (i.e., bilirubin and biliverdin) became dominant and were slowly removed as the bruise healed.

**Figure 5:**
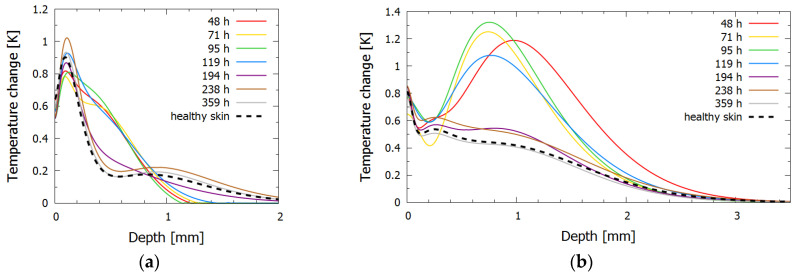
Inversely reconstructed initial temperature–depth profiles for bruise #2 for laser wavelengths (**a**) 532 nm and (**b**) 1064 nm. The profile for healthy skin is shown with a dashed line, and profiles for bruised skin at different times after the injury are shown in different colors.

In bruise #2, two distinct phases could be identified from the inversely reconstructed temperature profiles for both lasers, namely, the phase of persistent bruise and the phase of bruise removal. During the first five days (48–119 h), the 532 nm temperature profiles (Figure 5a) showed a noticeable deeper heat accumulation at the depth of approximately 0.5 mm. The measurement at 119 h showed the first signs of the bruise healing, as the amplitude of the heat accumulation peak started to decrease. The 1064 nm temperature profiles (Figure 5b) also exhibited two distinct phases, with signs of the bruise removal at 119 h. 

After 194 h (eight days) the extravasated hemoglobin was almost completely removed, and the temperature distribution approached that of the healthy skin for both wavelengths. The penultimate measurements (from 238 h to 359 h) corresponded almost exactly to the measurement of healthy skin, indicating that the bruise was completely resolved; nevertheless, the bruise was still visible in the photographs at those times as a yellow coloring of bilirubin, whose absorption peak is at 467 nm when diluted in albumin. As absorption of bilirubin is at 532 nm almost one-hundredth that at the peak and at 1064 nm negligible [39,40], both lasers used in our research could not probe this chromophore sufficiently.

Unlike in bruise #1, the epidermal peak was located deeper, which could be due to different factors, such as a slightly darker skin tone of the subject or the inability to distinguish the melanin and papillary dermis peaks in the presence of the papillary blood volume fraction increase.

### 3.4. Bruise #3—Inversely Reconstructed Initial Temperature–Depth Profiles

**Figure 6:**
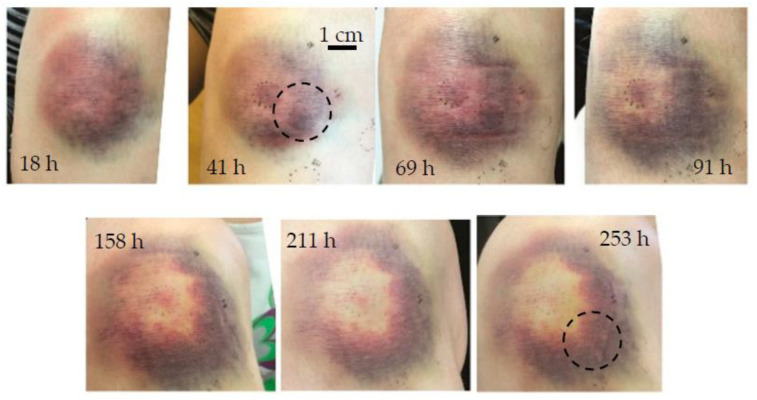
Chronological evolution of bruise #3. The circle indicates the area of measurement.

Bruise #3 occurred on the right knee of a 28-year-old female after a fall and represented a severe bruise (Figure 6). It was measured seven times between 18 h and 253 h (0.75 to 10.5 days) after injury.

In general, more severe bruising occurs on the knees as well as on the tibia or other parts of the human body where the bones lie close to the surface because the initial impact may press the tissue against the hard bone surface [41]. Early, pronounced bruising (as seen in Figure 6 at 18 h) was the first sign of the onset of severe hematoma; as expected, the effect of tissue swelling was also noticeable because the soft tissue layer was thinner, and any thickening of the tissue could be easily observed. The annular bruise shape that formed as the bruise healed could also be due to a small impact site that pushed the blood out of the impact area [13] or a more intense inflammatory reaction in the center [42]. The downward migration of the bruise, seen in Figure 6, was due to gravity and not because of lateral diffusion of hemoglobin.

**Figure 7:**
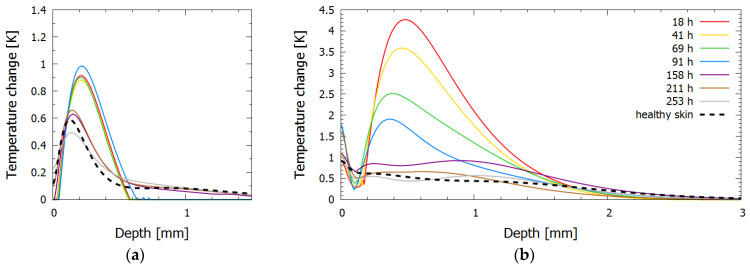
Inversely reconstructed initial temperature–depth profiles for bruise #3 for laser wavelengths (**a**) 532 nm and (**b**) 1064 nm.

The inversely reconstructed initial temperature–depth profiles (Figure 7) were consistent with the bruise severity evident in Figure 6. Here the 532 nm light was absorbed mainly in the first 0.5 mm of the skin. During the first four days (up to 91 h), there was almost no discernible difference between the subsequent temperature profiles (Figure 7a); moreover, the superficial temperature peak corresponding to epidermal melanin was absent. These features could be attributed to the fact that the 532 nm light was absorbed in the blood pool close to the epidermal–dermal junction and therefore did not penetrate the skin very deeply. After almost a week (at 158 h) the profile abruptly approached that of the healthy skin, indicating that the bruise was close to healing. 

In contrast, the 1064 nm profiles showed a continuous decrease in the accumulated heat in the dermis during the first week. Combined with the lack of the change in the 532 nm light absorption in this period, this could be due to the initial increase in tissue fluid because of considerable swelling and the resulting lymphatic fluid removal. In addition, the maximum temperature rise in bruise #3 was 4.2 °C, while in other bruises the maximum temperature rise did not exceed 1.5 °C for the same laser parameters and similar skin type.

### 3.5. Bruises #1 through #8—Integral under the Curve (IUC)

**Figure 8:**
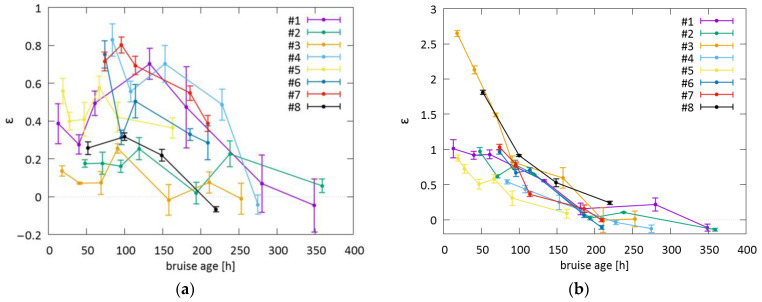
The ratio *ε* obtained for bruises #1 through #8 for (**a**) 532 nm and (**b**) 1064 nm lasers. The error bars represent standard deviations of *ε*.

For the 532 nm light (Figure 8a), the value of the relative change of IUC *ε* ranged from 20 to 80%. The maximum ratio was reached not in the first days after the injury but at later times, between 3 and 6 days, which indicated that hemoglobin required some time to diffuse from the blood pool to the surface. When the bruises resolved, the ratios approached 0. The severe bruise #3 showed the least prominent changes in ε, which could be attributed to the fact that the 532 nm light could not probe the deeper parts of the bruise, where active bruise healing and swelling were present.

For the 1064 nm light (Figure 8b), *ε* increased by 50 to 100% and up to 260% if swelling occurred as in bruises #3 and #8. During the first week (up to 168 h), there was generally a gradual and almost continuous decrease in ε (168 h). After approximately 200 h after the injury, the values approached 0, indicating the resolution of the bruise. The ability of the 1064 nm laser to assess a bruise decreased greatly after that time, as the bruise chromophores (blood and water) were replaced by degradation products, i.e., bilirubin, which could not be probed by the 1064 nm light. Bruise #3 (as well as bruise #8) exhibited different behavior with an extreme initial difference in *ε* in the first three days; such a deviation from other bruises could be attributed to the severity and swelling at the site of the bruise (Figure 6). Bruise #5, an example of a less severe contusion, exhibited the least change and the most rapid return to healthy skin.

## 4. Discussion

Our study illustrated the potential of PPTR profilometry to estimate the depths of bruises. The depth information is important for simulating the bruise evolution using bruise dynamics models [11,15,29,43]; moreover, research using colorimetry [6,7,10] would also benefit from the depth information as the color perception of the bruise is highly dependent on the depth of the extravasated blood, in addition to the skin tone or lighting conditions. While hyperspectral imaging (HSI) offers rough estimates of the depth [13], the addition of an independent technique to estimate the blood pool depth would most likely lead to more robust analyses of bruise dynamics.

The study results showed that the green light (532 nm) penetrated only a few hundred μm deep into tissue—some sources report the penetration depth of light in healthy fair skin to be about 300–350 μm [44], while others report a penetration depth of up to 900 μm [35]. Therefore, the KTP laser can mainly provide information on the shallower features of the bruised tissue. Since the main absorbers at this wavelength are melanin and hemoglobin [40], the PPTR signal includes primarily information on their superficial concentrations and depths. 

Near-infrared light (1064 nm) is reported to penetrate up to 3 mm [35] into the tissue. Our study results showed that this light penetrates approximately 1.6 mm. Here less light is absorbed in melanin and hemoglobin than with 532 nm light, but much more of the NIR light is absorbed in water, lipids, and collagen [40,45]. 

The feasibility of the PPTR technique for the bruise evolution research has already been assessed within our group [22,29,30,31,46]; however, only the 532 nm light has been used until now. Models of bruise evolution dynamics were developed with the goal to provide bruise age estimation. Adding a complementary wavelength to the models has the potential to allow more robust modeling of blood oxygenation and concentration if the absorption coefficients differ sufficiently between the chosen wavelengths. A wavelength where the water absorption is not negligible would also provide the ability to estimate water concentration and thus the presence of swelling, a characteristic physiological process especially in the first days after the bruise induction. However, the addition of an NIR wavelength requires significant modification of the bruise model by including important chromophores in the NIR spectral region, such as water, lipids, or collagen. The augmentation of the bruise model with the NIR wavelength data will be next step in our work.

In addition to PPTR, other noninvasive imaging techniques could also provide information about the location of the extravasated blood. Magnetic resonance imaging (MRI) can monitor magnetic changes in blood due to, e.g., blood clotting, oxygenation, and hemostasis and is therefore a viable technique for bruise research. Most of the research on bruising has been performed on bone bruises, and only a few studies have been performed on subcutaneous contusions [47] or on artificially produced hematomas [48]. However, these studies did not address the bruise depths and were also limited by the relatively low axial resolution of conventional MRI. High-resolution MRI [49] has adequate resolution (at 120 μm in the axial direction), but no such studies on bruises have been reported yet. Ultrasound (US) could in principle provide an estimate of the subcutaneous hemorrhage depth [50], with reported depths ranging from 1.6 to over 4 mm. However, Helm et al. [51] showed that bruises older than two days do not present sonographic changes, and as such, the technique may not be suitable for the study of older bruises. Optical coherence tomography (OCT), a popular optical technique for studying the superficial skin layers [52], has not, to our knowledge, been used to examine bruised skin. 

The time evolution of ε for both wavelengths showed interesting trends: a slow increase and decrease for the 532 nm laser and an almost monotonic decrease, with significant change in slope for the swelling phases, for the 1064 nm laser. The depths of the dermal absorption peaks for both wavelengths did not match, but this was to be expected because different tissue absorptions and scatterings result in substantially different penetration depths of the lasers. The 1064 nm laser profiles could also provide evidence of underlying changes, which are invisible to visible light, such as swelling of the tissue. This could prove useful in estimating the age of the bruise. The 1064 nm laser evaluation depth could slightly decrease in the case of swelling due to the excessive accumulation of interstitial fluid (approx. 5% increase [53]) and the corresponding increase in the absorption coefficient. Yet, the PPTR technique’s evaluation depth is intrinsically limited by the ill-posedness of the PPTR problem, which is the major limitation factor regarding the depth evaluation. Therefore, we do not expect that edema should decrease the evaluated depth. 

Additional data and more thorough analyses are needed to thoroughly study the potential differences in bruise dynamics. All subjects presented in this study were between 20 and 30 years of age and Caucasian (or type I–III on the Fitzpatrick scale), and only one was male. A larger sample size would be needed to characterize different bruise evolutions depending on subjects’ ages and sexes, skin colors, or bruise severities and locations. The latter could be achieved with a protocol for inducing bruises because it would then be possible to predetermine their location and the energy of the bruise-inducing projectile. A preliminary study of the induced bruises, approved by the national ethics committee, has already been performed in our research group [46] and will be extended in the future by involving more volunteers. 

The 532 nm laser PPTR measurement on dark-skinned and overly tanned individuals is inherently problematic because melanin becomes the main sink of absorbed photons. Therefore, photon accumulation occurs mainly outside of bruises, resulting in more noise in the signals and consequentially a loss of resolution. This drawback also applies to other optical techniques operating in the visible spectrum (DRS and HSI). However, at 1064 nm, the absorption in melanin decreases to 10%, the absorption in blood decreases to less than 2%, and the scattering in tissue decreases to less than 35% compared with 532 nm, while absorption in water increases more than 300-fold [45]. As such, the use of an NIR wavelength represents a potential workaround for the study of bruises in pigmented skin.

Unfortunately, the 532 and 1064 nm wavelengths are not sufficiently absorbed by the bruise breakdown product bilirubin. Therefore, older bruises in which all extravasated hemoglobin has been degraded to bilirubin do not appear in the PPTR signals or reconstructed initial temperature–depth profiles. As an extension of this study, a laser wavelength in the absorption range of bilirubin (450–500 nm) could be included to also provide information on bilirubin distribution. However, due to the high absorption in melanin and hemoglobin and the strong scattering in this spectral range, only the superficial bilirubin distribution could be investigated.

## 5. Conclusions

The combined 532 nm and 1064 nm PPTR temperature–depth profiling provided information on the depth distribution of the extravasated hemoglobin and other skin chromophore distributions in bruised skin in vivo. Specifically, the 532 nm laser had a moderate penetration depth of approx. 300–400 µm and probed mainly the superficial part of a bruise. The green laser provided information mainly about the melanin and the hemoglobin species distribution. The 1064 nm laser also probed deeper parts of a bruise up to approx. 1.6 mm, which was important for detection and monitoring of the blood pool typically located deeper in the bruised skin. Since the technique is noninvasive, it can be used for monitoring of bruise evaluation, showing how the extravasated blood diffuses through the skin and is being removed. In the future the two-wavelength PPTR approach can be used for the estimation of the bruise age, especially if combined with a model of bruise dynamics. 

## Figures and Tables

**Table 1:** Comparison of optical techniques used in bruise characterization.

	Colorimetry [7,33]	DRS [11]	HSI [15,34]	PPTR [30]
Measurement	L*a*b* color space values	Diffuse reflectance spectrum	Diffuse reflectance spectrum	Radiometric signal
Chromophore quantification	Skin color	All chromophore concentrations	All chromophore concentrations	Melanin and blood content
Lateral resolution	Point-wise measurement	Point-wise measurement	~0.1 mm	Point-wise measurement
Sensitivity to chromophore depth distribution	Poor	Poor	Poor	High

## Data Availability

The data that support the findings of this study are available upon reasonable request from the authors.

## Abbreviations

PPTR	pulsed photothermal radiometry
KTP	potassium titanyl phosphate
Nd:YAG	neodymium-doped yttrium aluminum garnet
DRS	diffuse reflectance spectroscopy
HSI	hyperspectral imaging
NIR	near-infrared
IUC	integral under curve
MRI	magnetic resonance imaging
US	ultrasound
OCT	optical coherence tomography

## References

[1] Langlois N.E. (2007). The science behind the quest to determine the age of bruises-a review of the English language literature. Forensic. Sci. Med. Pathol..

[2] Maguire S., Mann M., Sibert J., Kemp A. (2005). Can you age bruises accurately in children? A systematic review. Arch. Dis. Child..

[3] Grossman S., Johnston A., Vanezis P., Perrett D. (2011). Can we assess the age of bruises? An attempt to develop an objective technique. Med. Sci. Law.

[4] Pilling M., Vanezis P., Perrett D., Johnston A. (2010). Visual assessment of the timing of bruising by forensic experts. J. Forensic Leg. Med..

[5] Yajima Y., Funayama M. (2006). Spectrophotometric and tristimulus analysis of the colors of subcutaneous bleeding in living persons. Forensic Sci. Int..

[6] Scafide K., Sheridan D., Campbell J., DeLeon V., Hayat M. (2013). Evaluating change in bruise colorimetry and the effect of subject characteristics over time. Forensic Sci. Med. Pathol..

[7] Scafide K., Sheridan D., Taylor L., Hayat M. (2016). Reliability of tristimulus colourimetry in the assessment of cutaneous bruise colour. Inj.-Int. J. Care Inj..

[8] Mesli V., Le Garff E., Marchand E., Labreuche J., Ramdane N., Maynou C., Delannoy Y., Hedouin V. (2019). Determination of the age of bruises using a bilirubinometer. Forensic Sci. Int..

[9] Black H., Coupaud S., Daeid N., Riches P. (2019). On the relationships between applied force, photography technique, and the quantification of bruise appearance. Forensic Sci. Int..

[10] Hughes V., Langlois N. (2010). Use of reflectance spectrophotometry and colorimetry in a general linear model for the determination of the age of bruises. Forensic Sci. Med. Pathol..

[11] Randeberg L., Haugen O., Haaverstad R., Svaasand L. (2006). A novel approach to age determination of traumatic injuries by reflectance spectroscopy. Lasers Surg. Med..

[12] Sprigle S., Yi D., Caspall J., Linden M., Kong L., Duckworth M. (2007). Multispectral image analysis of bruise age. Proc. SPIE.

[13] Randeberg L., Larsen E., Svaasand L. (2010). Characterization of vascular structures and skin bruises using hyperspectral imaging, image analysis and diffusion theory. J. Biophotonics.

[14] Randeberg L., Hernandez-Palacios J., Kollias N., Choi B., Zeng H., Kang H., Knudsen B., Wong B., Ilgner J., Izdebski K. (2012). Hyperspectral Imaging of Bruises in the SWIR Spectral Region. Photonic Ther. Diagn. VIII Pts 1 2.

[15] Stam B., van Gemert M., van Leeuwen T., Teeuw A., van der Wal A., Aalders M. (2011). Can color inhomogeneity of bruises be used to establish their age?. J. Biophotonics.

[16] Hashemi S., Bahrani S., Mousavi S., Omidifar N., Behbahan N., Arjmand M., Ramakrishna S., Lankarani K., Moghadami M., Shokripour M. (2021). Ultra-precise label-free nanosensor based on integrated graphene with Au nanostars toward direct detection of IgG antibodies of SARS-CoV-2 in blood. J. Electroanal. Chem..

[17] Milner T., Goodman D., Tanenbaum B., Nelson J. (1995). Depth profiling of laser-heated chromophores in biological tissues by pulsed photothermal radiometry. J. Opt. Soc. Am. A-Opt. Image Sci. Vis..

[18] Majaron B., Sustercic D., Lukac M., Skaleric U., Funduk N. (1998). Heat diffusion and debris screening in Er: YAG laser ablation of hard biological tissues. Appl. Phys. B-Lasers Opt..

[19] Majaron B., Plestenjak P., Lukac M. (1999). Thermo-mechanical laser ablation of soft biological tissue: Modeling the micro-explosions. Appl. Phys. B-Lasers Opt..

[20] Majaron B., Verkruysse W., Tanenbaum B., Milner T., Telenkov S., Goodman D., Nelson J. (2000). Combining two excitation wavelengths for pulsed photothermal profiling of hypervascular lesions in human skin. Phys. Med. Biol..

[21] Fomina P., Proskurnin M. (2022). Photothermal radiometry methods in materials science and applied chemical research. J. Appl. Phys..

[22] Marin A., Verdel N., Milanič M., Majaron B. (2021). Noninvasive Monitoring of Dynamical Processes in Bruised Human Skin Using Diffuse Reflectance Spectroscopy and Pulsed Photothermal Radiometry. Sensors.

[23] Milanic M., Sersa I., Majaron B. (2009). A spectrally composite reconstruction approach for improved resolution of pulsed photothermal temperature profiling in water-based samples. Phys. Med. Biol..

[24] Wang T., Mallidi S., Qiu J., Ma L., Paranjape A., Sun J., Kuranov R., Johnston K., Milner T. (2011). Comparison of pulsed photothermal radiometry, optical coherence tomography and ultrasound for melanoma thickness measurement in PDMS tissue phantoms. J. Biophotonics.

[25] Milanic M., Majaron B., Nelson J. (2007). Pulsed photothermal temperature profiling of agar tissue phantoms. Lasers Med. Sci..

[26] Verdel N., Marin A., Milanic M., Majaron B. (2019). Physiological and structural characterization of human skin in vivo using combined photothermal radiometry and diffuse reflectance spectroscopy. Biomed. Opt. Express.

[27] Jacques S., Nelson J., Wright W., Milner T. (1993). Pulsed photothermal radiometry of port-wine-stain lessions. Appl. Opt..

[28] Milner T., Smithies D., Goodman D., Lau A., Nelson J. (1996). Depth determination of chromophores in human skin by pulsed photothermal radiometry. Appl. Opt..

[29] Vidovic L., Milanic M., Randeberg L., Majaron B., Vitkin A., Amelink A. (2013). Characterization of the bruise healing process using pulsed photothermal radiometry. Nov. Biophotonic Tech. Appl. II.

[30] Vidovic L., Milanic M., Majaron B. (2015). Objective characterization of bruise evolution using photothermal depth profiling and Monte Carlo modeling. J. Biomed. Opt..

[31] Marin A., Verdel N., Vidovič L., Milanič M., Majaron B. Assessment of individual bruising dynamics by pulsed photothermal radiometry and inverse Monte Carlo analysis. Proceedings of the European Conference on Biomedical Optics.

[32] Milanic M., Majaron B. (2013). Energy deposition profile in human skin upon irradiation with a 1,342 nm Nd:YAP laser. Lasers Surg. Med..

[33] Ly B., Dyer E., Feig J., Chien A., Del Bino S. (2020). Research Techniques Made Simple: Cutaneous Colorimetry: A Reliable Technique for Objective Skin Color Measurement. J. Investig. Dermatol..

[34] Stergar J., Hren R., Milanic M. (2022). Design and Validation of a Custom-Made Laboratory Hyperspectral Imaging System for Biomedical Applications Using a Broadband LED Light Source. Sensors.

[35] Bashkatov A., Genina E., Kochubey V., Tuchin V. (2005). Optical properties of human skin, subcutaneous and mucous tissues in the wavelength range from 400 to 2000 nm. J. Phys. D-Appl. Phys..

[36] Guyton A., Hall J. (2006). Textbook of Medical Physiology.

[37] Langlois N., Gresham G. (1991). The ageing of bruises: A review and study of the colour changes with time. Forensic Sci. Int..

[38] Vargas O., Chan E., Barton J., Rylander H., Welch A. (1999). Use of an agent to reduce scattering in skin. Lasers Surg. Med..

[39] Kanick S., van der Leest C., Aerts J., Hoogsteden H., Kascakova S., Sterenborg H., Amelink A. (2010). Integration of single-fiber reflectance spectroscopy into ultrasound-guided endoscopic lung cancer staging of mediastinal lymph nodes. J. Biomed. Opt..

[40] Bydlon T., Nachabe R., Ramanujam N., Sterenborg H., Hendriks B. (2015). Chromophore based analyses of steady-state diffuse reflectance spectroscopy: Current status and perspectives for clinical adoption. J. Biophotonics.

[41] Cox W.A. Pathology of Blunt Force Traumatic Injury. https://forensicmd.files.wordpress.com/2011/05/blunt-force-traumatic-injuries.pdf.

[42] Barington K., Skovgaard K., Henriksen N., Johansen A., Jensen H. (2018). The intensity of the inflammatory response in experimental porcine bruises depends on time, anatomical location and sampling site. J. Forensic Leg. Med..

[43] Stam B., van Gemert M., van Leeuwen T., Aalders M. (2012). How the blood pool properties at onset affect the temporal behavior of simulated bruises. Med. Biol. Eng. Comput..

[44] Mustafa F., Jaafar M. (2013). Comparison of wavelength-dependent penetration depths of lasers in different types of skin in photodynamic therapy. Indian J. Phys..

[45] Jacques S. (2013). Optical properties of biological tissues: A review. Phys. Med. Biol..

[46] Marin A., Milanič M., Verdel N., Vidovič L., Majaron B. (2018). Dynamics of controllably induced bruises assessed by diffuse reflectance spectroscopy and pulsed photothermal radiometry. Proc. SPIE.

[47] Langlois N., Ross C., Byard R. (2013). Magnetic resonance imaging (MRI) of bruises: A pilot study. Forensic Sci. Med. Pathol..

[48] Hassler E., Ogris K., Petrovic A., Neumayer B., Widek T., Yen K., Scheurer E. (2015). Contrast of artificial subcutaneous hematomas in MRI over time. Int. J. Leg. Med..

[49] Barral J., Bangerter N., Hu B., Nishimura D. (2010). In Vivo High-Resolution Magnetic Resonance Skin Imaging at 1.5 T and 3 T. Magn. Reson. Med..

[50] Mimasaka S., Oshima T., Ohtani M. (2012). Characterization of bruises using ultrasonography for potential application in diagnosis of child abuse. Leg. Med..

[51] Helm T., Bir C., Chilstrom M., Claudius I. (2016). Ultrasound characteristics of bruises and their correlation to cutaneous appearance. Forensic Sci. Int..

[52] Olsen J., Holmes J., Jemec G. (2018). Advances in optical coherence tomography in dermatology-a review. J. Biomed. Opt..

[53] Eikje N., Ozaki Y., Aizawa K., Arase S. (2005). Fiber optic near-infrared Raman spectroscopy for clinical noninvasive determination of water content in diseased skin and assessment of cutaneous edema. J. Biomed. Opt..

